# Effect of vitamin C fortification on the quality of cow's and goat's yoghurt

**DOI:** 10.1002/fsn3.2959

**Published:** 2022-07-18

**Authors:** Alicja Sobczak, Marzena Danowska‐Oziewicz, Katarzyna Ząbek, Jan Miciński, Agnieszka Narwojsz

**Affiliations:** ^1^ Department of Sheep and Goat Breeding, Faculty of Animal Bioengineering University of Warmia and Mazury in Olsztyn Olsztyn Poland; ^2^ Department of Human Nutrition, Faculty of Food Sciences University of Warmia and Mazury in Olsztyn Olsztyn Poland

**Keywords:** acerola, L‐ascorbic acid, milk product, pH, sensory evaluation

## Abstract

Yoghurt is one of the well‐known fermented dairy products that play an important role in the human diet. At present, products made of goat's milk are becoming more popular. This study was conducted to evaluate the effect of physicochemical properties of yoghurt fortified with vitamin C. Six different yoghurts were developed: from goat's and cow's milk without any addition, with L‐ascorbic acid and acerola addition. The results showed that the addition of L‐ascorbic acid significantly decreased pH. Based on the sensory evaluation, the natural cow's yoghurt has scored higher in the overall rating among yoghurts. The addition of L‐ascorbic acid to natural goat's yoghurt positively affected the color, taste, flavor, and consistency. In the case of cow's milk yoghurt, the addition of L‐ascorbic acid and acerola deteriorated the taste of the product.

## Graphical abstract

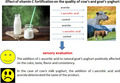

## 1 INTRODUCTION

Yoghurt is one of the oldest fermented dairy products worldwide and still plays an important role in the human diet today due to its pleasant taste and health benefits (Chen et al., 2017). This product is a coagulated milk product made by fermenting lactose in milk by lactic acid bacteria: *Streptococcus thermophilus* (*S. thermophilus*) and *Lactobacillus delbrueckii ssp. bulgaricus* (*Lb. bulgaricus*) to liberate lactic acid which is responsible for its unique taste (Rul, 2017). Many products are enriched with the addition of probiotic bacteria of the genera *Bifidobacterium* and *Lactococcus* (Voidarou et al., 2021). Yoghurt is considered to be a healthy food because of its high digestibility and bioavailability of nutrients. It can also be recommended to the people with lactose intolerance, gastrointestinal disorders such as enteritis and irritable bowel disease, and supports immune function and weight control (Weerathilake et al., 2014). Most yoghurts are made from cow's milk. Nowadays, there is a growing interest in the development of new dairy products, including yoghurts, by substituting cow's milk with other mammals' milk (Dimitrellou et al., 2019). This trend can be attributed to the yoghurt's nutritional and therapeutic value as well as beneficial features of other types of milk. Goat's milk is characterized by different physicochemical properties compared to cow's milk, and this may be attributed to smaller size of fat globules and a higher percentage of short‐ and medium‐chain fatty acids (Lima et al., 2018). According to Kumar and Sharma (2016), goat's milk is more similar to human milk than cow's milk in terms of protein structure and oligosaccharide profile, shows better digestibility and absorption, and causes lower allergenicity than cow's milk. The bioavailability of minerals in goat's milk is also higher than in cow's milk (Turkmen, 2017). Goat's and cow's milk are both deficient in pyridoxine (B_6_), vitamins C and D (Kumar et al., 2012).

Fortified foods are enriched with one or more essential nutrients for the purpose of preventing or treating a demonstrated deficiency in that nutrient. Yoghurt may be fortified with minerals and vitamins, among others, especially with vitamin C. Vitamin C (L‐ascorbic acid) contributes to immune defense and has been known as an antioxidant. It is necessary for the proper growth and development of the organism, plays a role in several physiological processes, such as immune stimulation, biosynthesis of collagen, hormones, neurotransmitters, iron absorption, and is an essential enzyme cofactor. Vitamin C supports epithelial barrier function against pathogens and accumulates in phagocytic cells, such as neutrophils. It is also needed for apoptosis and removal of the spent neutrophils from sites of infection by macrophages (Doseděl et al., 2021). Significant vitamin C deficiency causes scurvy, while limited vitamin C intake causes symptoms such as increased susceptibility to infections, loosening of the teeth, dryness of mouth and eyes, hair loss, dry itchy skin, fatigue, and insomnia (Pehlivan, 2017). Plants and most animals synthesize ascorbate from glucose, while primates, including humans, cannot synthesize ascorbate (Moritz et al., 2020). Dietary Reference Intake (DRI) recommends 90 mg/day of vitamin C for adult men and 75 mg/day for adult women, and no more than 2 g (2.000 mg) per day. Some populations need special attention of vitamin C requirements. These include patients with periodontal disease, smokers, pregnant and lactating women, and the elderly. According to DRI, the recommendation for women during pregnancy and lactation is 85 mg/day and 120 mg/day of vitamin C, respectively (DRI, 2000). Vitamin C, in addition to its impact on humans, also serves as a reagent for the preparation of many materials in the pharmaceutical and food industries. The synthetic vitamin C is used as a food additive (E300), as it can extend food durability (Silva & Lidon, 2016).

One of the possible vitamin C supplementation options is the addition of fruit to yoghurt. The fruits are compatible with dairy products. The beneficial synergy between these products and the bioactive ingredients identified in dairy products has the potential to lead to a new era in functional food innovation (Guiné & De Lemos, 2020). Acerola fruits are well known for their high vitamin C content as well as phenolic compounds including benzoic acid derivatives, phenylpropanoids, flavonoids, and carotenoids (Belwal et al., 2018). According to Lima et al. (2005), the content of vitamin C in acerola is high, and might vary from 1247.10 to 1845.79 mg/100 g. The presence of numerous bioactive compounds in acerola determines its functional and prohealthy properties such as hyperglycemic, antihyperlipidemic, anti‐photoaging, anti‐inflammatory, hepatoprotective, antitumor, anti‐obesity, antioxidant, and antimicrobial, among the others (Belwal et al., 2018), and makes it a potential source of valuable components in the food industry. The processing and production of new food types can significantly affect the quality and properties of bioactive fruit compounds, and the study of these effects is of fundamental and practical importance to optimize processes and improve yoghurt quality (Cușmenco & Bulgaru, 2020).

The aim of the present study was to investigate the selected physicochemical properties of yoghurts made from cow's and goat's milk with the addition of L‐ascorbic acid or acerola fruit extract (*Malpighia glabra*).

## 2 MATERIAL AND METHODS

The yoghurts made of goat's and cow's milk were used as a research material in the present study. The yoghurt samples were manufactured under laboratory conditions, using milk with the addition of lactic acid bacteria cultures. Both the goat's and cow's milk were first pasteurized at a temperature of 70 to 72°C for 25 min and then cooled to 40°C. For the yoghurts' manufacture, the YO‐122 starter culture (SEROWAR s.c., Szczecin, Poland), which contained *Streptococcus salivarius subsp. thermophilus* and *Lactobacillus delbrueckii subsp. bulgaricu*s, was used for inoculation. The amount of the inoculant was 1% in relation to the weight of the milk. The inoculated milk was kept at temperature 42 ± 2°C for 6 h until a pH of 4.6 was reached. Each yoghurt (goat's and cow's milk product, coded G and C, resp.) was divided into 3 samples. The samples coded G‐C_1_ and C‐C_1_ were left without additives and used as a control group. Powdered acerola (G‐S_2_ and C‐S_2_) or L‐ascorbic acid (G‐S_3_ and C‐S_3_) was added to the experimental samples. Supplements were added at the ratios of 18 mg/100 g of L‐ascorbic acid (Vitamin C, CZDA, Stanlab®, Lublin, Poland) and 72 mg/100 g of acerola (*Malpighia glabra* powder, Sanbios®, Gliwice, Poland). The amount of acerola added was due to the vitamin C content (1 g acerola = 250 mg vitamin C).

The samples of yoghurt were analyzed for chemical parameters (dry matter, protein and fat content). Additionally, for all samples, pH, vitamin C content, and sensory evaluation parameters (flavor aroma, appearance, color, overall acceptability) were analyzed. The dry matter of the yoghurts was determined according to the Association of Official Analytical Chemists method (AOAC, 2005). Each yoghurt sample (10 g) was placed in a laboratory oven (UF55, Memmert, Schwabach, Germany) at 105°C and dried to a constant weight. Protein content was analyzed by the method of Lowry et al. (1951). Fat content was determined in the butyrometer by the Gerber method (BS 696‐1:^1955^), modified according to Fahmid et al. (2016). The pH values were measured using a digital pH‐meter (HI 2211, Hanna Instruments, Leighton Buzzard, UK). Vitamin C content was determined using the titrimetric 2,6‐dichlorophenolindophenol method. The principle of the method was the extraction of the ascorbic acid in the test sample with 2% oxalic acid solution and titrating with 2,6‐dichlorophenolindophenol until a light pink color was developed (PN‐A‐04019:^1998^). All analyses were carried out in triplicate.

Sensory evaluation was performed by a panel of 50 judges, consisting of 10 members of the university staff and 40 untrained students. The assessment panel evaluated the selected sensory characteristics of the samples using a 5‐point hedonic scale according to Ogden (1993) ranging from very poor (score = 1) to excellent (score = 5) as extremes. Tests were conducted in a sensory analysis laboratory equipped with five individual booths. Samples were served in white plastic containers identified with three‐digit code number. Each sample was evaluated by the assessor with three repeats (Baryłko‐Pikielna & Matuszewska, 2009).

The results of the study were presented as mean values with standard deviations (*SD*s) in the tables and only mean values in the figures. The results were processed statistically by the analysis of variance (ANOVA) test for factorial designs, and the significance of differences between means in groups was verified by the Duncan's test, using Statistica 13.0 software (TIBCO Software Inc., Tulsa, OK, USA).

## 3 RESULTS AND DISCUSSION

The comparison of the chemical composition of goat's and cow's yoghurt is presented in Table 1. The results show that goat's yoghurt had a slightly higher content of dry matter, protein, and fat, however the differences were not statistically significant. The result agrees with the statement of Temerbayeva et al. (2018) who developed the goat's yoghurt that contained 2.84% protein and 4.17% fat and cow's yoghurt only 2.66%, 3.55%, respectively. Nevertheless, in that study the initial values for basic components were higher in goat's milk than in cow's milk used for yoghurts' manufacture and therefore the difference in yoghurts' composition was rather a result of difference in milk composition. Ehirim and Onyenek (2013) reported that goat's yoghurt contains more protein (4.27%) compared to 3.22% in cow's milk yoghurt. The fat and protein content of goat's yoghurt was higher in studies carried out by Cușmenco and Bulgaru (2020) than in our experiment. In the present research, fat content of the goat's yoghurt was lower but protein content was higher than that reported by Amal et al. (2016), Güler and Şanal (2009), and Kesenkaş et al. (2017). Considering a similar basic composition of cow's and goat's yoghurts observed in our study, it is worth pointing out another advantage of goat's dairy products. The smaller fat globules and lack of agglutinin in goat's milk compared to cow's milk make it a valuable material for the dairy industry, as both these features allow milk to stay homogeneous and eliminate the necessity of milk homogenization and various problems related to this process (Kumar & Sharma, 2016).

**TABLE 1:** Results of basic chemical composition analysis of yoghurts (%)

Traits	Group					
	Cow's yoghurt			Goat's yoghurt		
	C‐C_1_	C‐S_2_	C‐S_3_	G‐C_1_	G‐S_2_	G‐S_3_
Dry matter	12.98 ± 0.21	12.99 ± 0.56	12.97 ± 0.33	13.06 ± 0.23	13.07 ± 0.57	13.04 ± 0.45
Crude protein	4.09 ± 0.22	4.11 ± 0.18	4.08 ± 0.16	4.54 ± 0.21	4.56 ± 0.25	4.53 ± 0.23
Crude fat	2.20 ± 0.08	2.21 ± 0.01	2.21 ± 0.10	2.93 ± 0.14	2.93 ± 0.04	2.91 ± 0.19

*Note*: No statistically significant differences were found between yoghurts at *p* ≤ .05 and *p* ≤ .01.

**FIGURE 1:**
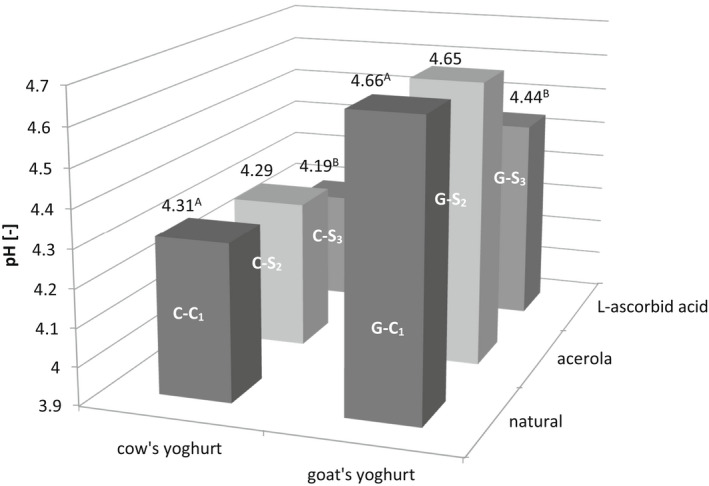
Changes of pH values in analyzed yoghurts (A, B—Mean values with different letters within a yoghurt type differ significantly at *p* ≤ .01)

The above results show that the addition of L‐ascorbic acid and powdered acerola did not affect the chemical composition of investigated yoghurts since no significant differences were found in dry matter, total protein, and fat content between these types of yoghurt. The addition of acerola and L‐ascorbic acid to yoghurts influenced their acidity (Figure 1). Lower pH values of both cow's and goat's yoghurts with the addition of L‐ascorbic acid compared to the respective control samples were observed, while there were no significant differences between yoghurts with acerola and respective control products. This observation may suggest better stability of the pH value of yoghurt enriched with acerola than with vitamin C, presumably due to the buffering capacity of numerous other compounds present in acerola that contributed to the natural buffering capacity of yoghurt (Belwal et al., 2018; Salaün et al., 2005). The pH values of cow's yoghurts recorded in the present study were lower than the results obtained by Amal et al. (2016), Cho et al. (2020), and Dimitrellou et al. (2020). Teichert et al. (2015) showed that the pH of goat's yoghurt ranged from 4.61 to 4.63 depending on the bacterial cultures used in the experiment. Contrary results were obtained by Sigdel et al. (2018), who reported that the addition of dried mulberry (9.6 mg % of vitamin C) to yoghurt significantly reduced the pH from 4.37 to 4.31.

**FIGURE 2:**
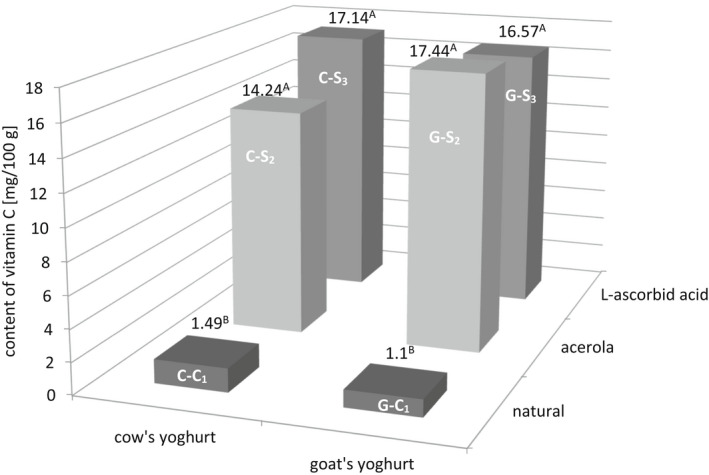
The content of vitamin C in yoghurt; (A, B—Mean values with different letters within a yoghurt type differ significantly at *p* ≤ .01)

The content of ascorbic acid in investigated yoghurts ranged from 1.10 to 17.44 mg/100 g (Figure 2). The data show that the yoghurts enriched with vitamin C significantly increased its concentration in samples, regardless of the source of this compound (*p* ≤ .01). The highest content of vitamin C was noticed in the goat's yoghurt with acerola (17.44 mg/100 g), followed by the cow's yoghurt with L‐ascorbic acid (17.14 mg/100 g). Within a yoghurt type (cow's or goat's products), samples with acerola or L‐ascorbic acid added did not differ significantly in terms of vitamin C content. The amount of acerola added was intended in such amount to provide the same amount of vitamin C as it was added in the form of L‐ascorbic acid.

Sensory attributes of color, aroma, taste, consistency, and overall rating, for all yoghurt formulations, are displayed in Table 2. It can be seen that generally higher scores were obtained by the cow's yoghurts. The highest overall quality grade—4.70 points was given to the control cow's yoghurt. This yoghurt received the best scores for most attributes, except consistency, among the evaluated samples. The color was rated the highest in natural cow's yoghurt and cow's yoghurt with L‐ascorbic acid, while the lowest score was noted for goat's yoghurt with acerola. Goat's yoghurt with L‐ascorbic acid showed an improved color compared with the control goat's yoghurt. The addition of acerola to the yoghurts changed the color of these milk products from white to light pink. This characteristic was apparently perceived as a less attractive sensory property (color) by the panelists. The control cow's yoghurt was rated the highest in terms of taste (*p* ≤ .01). The scores for consistency and overall rating indicate that the control cow's yoghurt, cow's yoghurts with L‐ascorbic acid and acerola did differ significantly in terms of these features (scores above 4 points), while the consistency of goat's yoghurts, especially that with acerola, was rated significantly lower (*p* ≤ .01). The results of flavor evaluation followed the same trend. Products with acerola received the lowest ratings for almost all evaluated attributes in both yoghurt groups.

**TABLE 2:** Results of the sensory assessment of yoghurts (points)

Traits	Group					
	Cow's yoghurt			Goat's yoghurt		
	C‐C_1_	C‐S_2_	C‐S_3_	G‐C_1_	G‐S_2_	G‐S_3_
Color	5.00 ± 0.01^Aa^	4.30 ± 1.25^a^	5.00 ± 0.01^Aa^	4.00 ± 0.82^b^	3.70 ± 1.16^B^	4.10 ± 0.74^b^
Taste	4.70 ± 0.48^A^	3.50 ± 0.97^B^	3.70 ± 0.67^Ba^	2.70 ± 0.82^Bb^	2.80 ± 1.14^Bb^	3.40 ± 0.52^B^
Flavor	4.50 ± 0.71^Aa^	4.50 ± 0.97^Aa^	4.50 ± 0.71^Aa^	3.20 ± 0.79^B^	3.10 ± 1.29^B^	3.50 ± 1.18^b^
Consistency	4.70 ± 0.67^A^	4.40 ± 1.26^A^	4.90 ± 0.32^A^	2.30 ± 1.06^B^	2.00 ± 1.05^B^	2.40 ± 0.97^B^
Overall rating	4.70 ± 0.41^A^	4.20 ± 0.67^A^	4.58 ± 0.32^A^	2.96 ± 0.70^B^	2.86 ± 1.04^B^	3.28 ± 0.77^B^

*Note*: A, B—mean values in rows with different letters differ significantly at *p* ≤ .01. a, b—mean values in rows with different letters differ significantly at *p* ≤ .05.

Generally, cow's yoghurt samples were scored significantly higher than goat's yoghurts. The results are in agreement with the statement of Temerbayeva et al. (2018) who showed that taste, appearance, color, and flavor were improved by combination of goat and cow milk compared to the yoghurt made from only goat milk. Opposite observations were noted by Ehirim and Onyenek (2013), who reported that sensory evaluation results of goat's yoghurt samples were higher for taste, aroma, consistency, and general acceptance than those observed for samples of cow's yoghurt.

## 4 CONCLUSIONS

The current study supports the conclusion that the addition of vitamin C to yoghurt produces an enriched yoghurt with desirable properties compared to plain yoghurt. The addition of L‐ascorbic acid to natural goat's yoghurt positively affected the color, taste, flavor, and consistency. In the case of cow's milk yoghurt, the addition of L‐ascorbic acid and acerola deteriorated the taste of the product. Consumption of fortified yoghurt might be a healthy daily diet supplement. Our results show that yoghurt enriched with vitamin C by adding acerola—its natural source—received similar scores for sensory traits to the controls, and they suggest that acerola seems to be quite effective as an additive particularly to the goat's yoghurt. There is a strong tradition of cow's milk products' consumption in Poland, however goat's milk products (mainly soft cheese) are also present on the market. Availability of goat's yoghurts with acerola on the market may be an interesting alternative for health‐oriented consumers, offering them benefits of goat milk, yoghurts, and valuable bioactive compounds of acerola.

## FUNDING INFORMATION

The Project was financially supported by the Ministry of Education and Science in the range of the program entitled “Regional Initiative of Excellence” for the years 2019–2022, Project No. 010/RID/2018/19, amount of funding 12.000.000 PLN.

## CONFLICT OF INTEREST

The authors declare no conflict of interest.
